# Histology of Convergent Probing Appendages in Mormyridae

**DOI:** 10.1093/iob/obad001

**Published:** 2023-01-20

**Authors:** R D Peterson, A J Evans, L P Hernandez

**Affiliations:** Department of Biological Sciences, The George Washington University, Washington DC 20052, USA; Department of Biological Sciences, The George Washington University, Washington DC 20052, USA; Department of Biological Sciences, The George Washington University, Washington DC 20052, USA

## Abstract

Mormyridae is an early diverging family of Teleostean fishes that produce an electric field for navigation and communication using an electric organ. This clade has a diverse array of soft-tissue rostral appendages, such as the chin-swelling, the Schnauzenorgan, and the tubesnout combined with a Schnauzenorgan, that have evolved multiple times. Here we assess if macroscopically convergent, soft-tissue rostral appendages are also histologically convergent. Further, we investigate how the histology of these appendages can inform their function. We sampled independent gains of the chin-swelling and Schnauzenorgan to understand similarities and differences in their anatomies. We show that macroscopically convergent rostral appendages are also convergent at a histological level, and different types of rostral appendages share a similar anatomy; that said, minor differences likely relate to their specific functions. Based on a comparison of the skeletal muscle distribution and the differing attachment shapes of each appendage to the dentary, we conclude that the Schnauzenorgan is capable of a wider range of movements than the chin swelling. Furthermore, the anatomy suggests that these soft-tissue rostral appendages likely function as electrosensory foveas (i.e., an appendage that focuses a sensory system). Lastly, these histological data support the hypothesis that the chin swelling may be a precursor to the Schnauzenorgan.

## Abstract (es)

Resumen

Mormyridae es una de las primeras familias divergentes de peces teleósteos que producen un campo eléctrico para la navegación y la comunicación mediante un órgano eléctrico. Este clado tiene una variedad diversa de apéndices rostrales de tejidos blandos, como el mentón hinchado, el Schnauzenorgan y el hocico tubular combinado con un Schnauzenorgan, que han evolucionado varias veces. Aquí evaluamos si los apéndices rostrales de tejido blando macroscópicamente convergentes también son histológicamente convergentes. Además, investigamos cómo la histología de estos apéndices puede informar sus funciones. Tomamos muestras de ganancias independientes del mentón hinchado y del Schnauzenorgan para comprender las similitudes y diferencias en sus anatomías. Mostramos que los apéndices rostrales macroscópicamente convergentes también lo son a nivel histológico, y diferentes tipos de apéndices rostrales comparten una anatomía similar; dicho esto, es probable que las diferencias menores se relacionen con sus funciones específicas. Con base en una comparación de la distribución del músculo esquelético y las diferentes formas de unión de cada apéndice al dentario, concluimos que el Schnauzenorgan es capaz de realizar una gama más amplia de movimientos que el mentón hinchado. Además, la anatomía sugiere que estos apéndices rostrales de tejido blando probablemente funcionen como fóveas electrosensoriales (es decir, un apéndice que enfoca un sistema sensorial). Por último, estos datos histológicos apoyan la hipótesis de que el mentón hinchado puede ser un precursor del Schnauzenorgan.

## Introduction

**Fig. 1:**
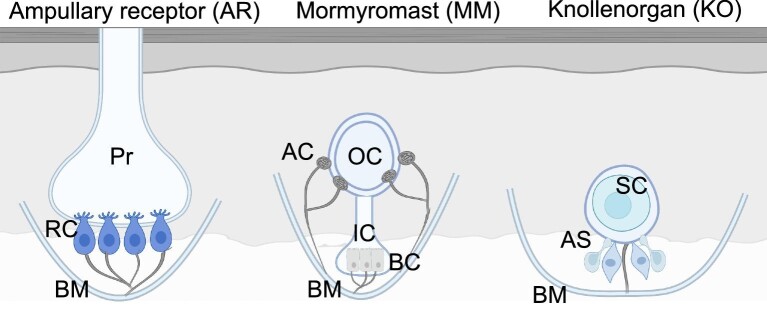
Three different types of electroreceptors in Mormyridae. The ampullary receptor: Pr, pore; RC, receptor cells; BM, basement membrane. Mormyromast: OC, outer chamber; AC, A-cells; IC, inner chamber, BC, B-cells; BM, basement membrane. Knollenorgan: SC, sensory cell; AS, accessory cells; BM, basement membrane. Diagram based on C. C. Bell et al., 1989; Kawasaki, 1970; Szabo, 1974.

Mormyrids, the most diverse family of osteoglossomorphs (bonytongues), are well known for their ability to hunt, communicate, and navigate within their surroundings using an electric organ that produces an electric field. Mormyrids are capable of both passive and active electrolocation (Bullock 1973; Bullock and Heiligenberg 1986; Heiligenberg 1987; Bastian 1990; Von Der Emde et al. 1998; Albert and Crampton 2005). Passive electrolocation is facilitated by ampullary receptors that locate objects or prey by detecting muscle action potentials and transmembrane potentials (Crampton 2019) (Fig. 1). Alternatively, active electrolocation uses tuberous electroreceptors to detect distortions of individual electric fields produced by an electric organ (Lissmann 1958). Two types of tuberous electroreceptors characterize mormyrids. The first, mormyromasts (Fig. 1), are amplitude-coding receptors that are used in active electrolocation and are responsible for detecting self-generated electric organ discharges (EODs). These receptors are commonly found on appendages that project from the chin/lower jaw, henceforth called rostral appendages. The second, knollenorgans (Fig. 1), are time-coding receptors that detect species- and sex-specific EODs and are used exclusively in communication. Compared to mormyromasts, knollenorgans can detect a broader range of frequencies and are more sensitive (Bullock and Heiligenberg 1986; Von Der Emde et al. 1998; Bell and Maler 2005; Crampton 2019). While abundant research has been conducted on their electrophysiology, the morphological diversity of rostral appendages in mormyrids has been largely overlooked.

**Fig. 2:**
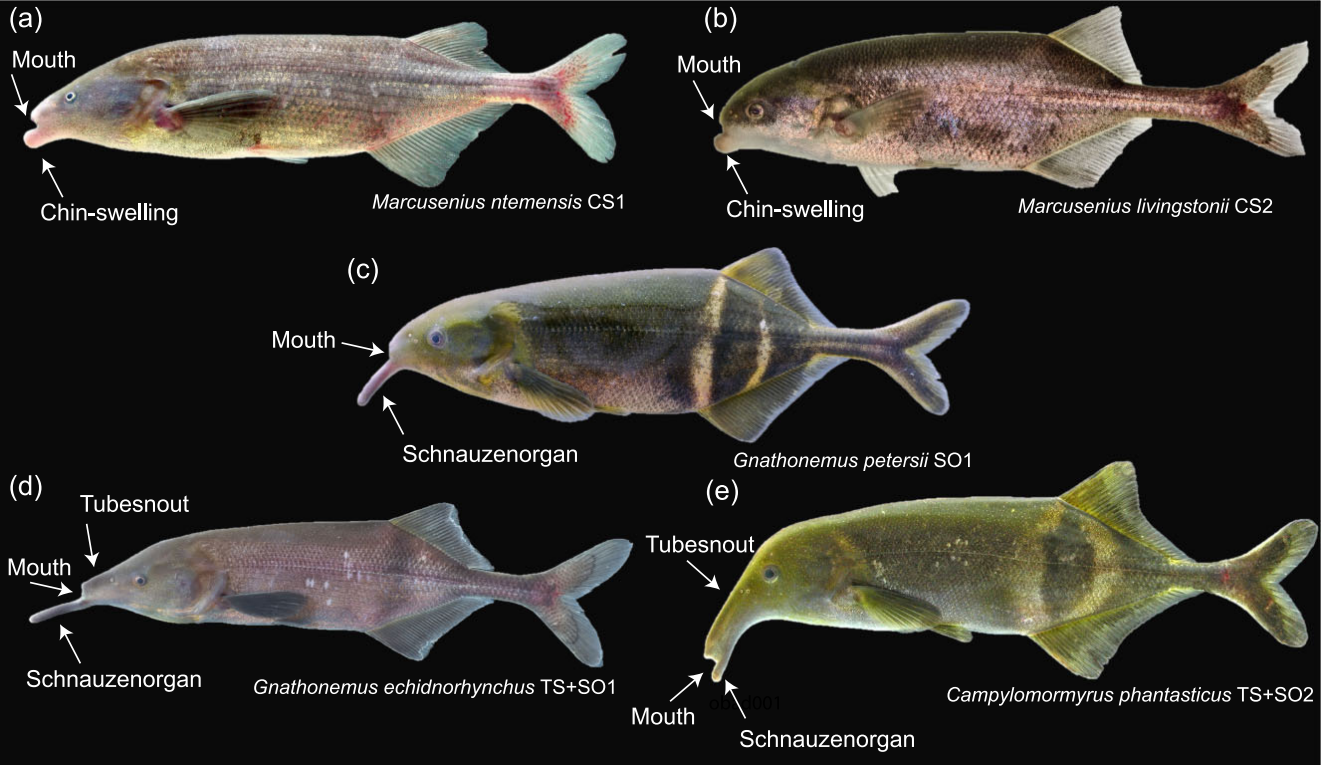
The craniofacial morphologies present within the mormyrids examined. CS1 = Chin-swelling gain 1; CS2 = Chin-swelling gain 2; SO1 = Schnauzenorgan gain 1; TS+SO1= tubesnout with Schnauzenorgan gain 1; TS+SO2= tubesnout with Schnauzenorgan gain 2 (A) The first independent gain of the chin-swelling is represented by *Marcusenius ntemensis*. (B) The second independent gain of the chin-swelling is represented by *Marcusenius livingstonii*. (C) The single gain of the Schnauzenorgan is represented by *Gnathonemus petersii*. (D) The first independent gain of the tubesnout with a Schnauzenorgan is represented by *Gnathonemus echidnorhynchus*. (E) The second independent gain of the tubesnout with a Schnauzenorgan is represented by *Campylomormyrus phantasticus*. Photo credits: John P Sullivan.

**Fig. 3:**
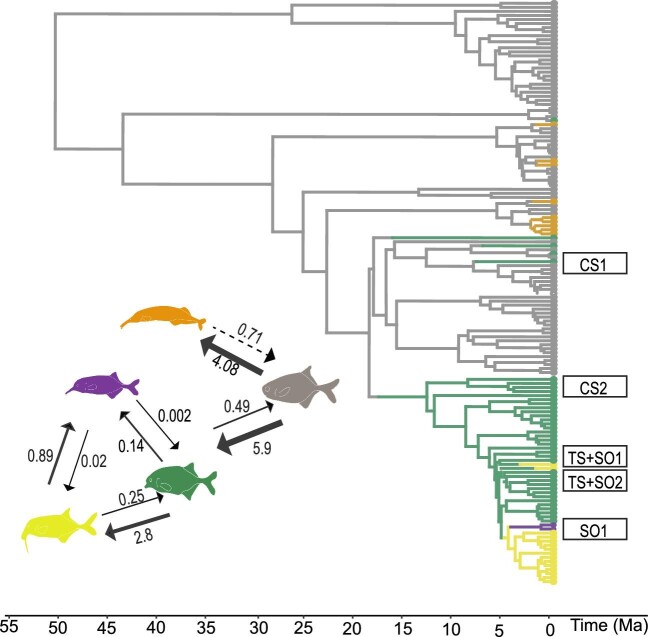
Ancestral state reconstruction of different craniofacial morphologies present within mormyrids. Branch colors represent different character states: gray is blunt-nosed character state, green is the chin-swelling state, purple is the Schnauzenorgan and yellow is the tubesnout with a Schnauzenorgan. The circles at the tip of the tree represent the character state that is present in each mormyrid. The labels to the right of the tree tips indicate which branches were sampled for this study. The cartoon fishes to the left of the tree represent the number of times each character state was inferred in the ancestral state estimation. This figure is adapted from results in Peterson et al., 2022.

Fishes within the family Mormyridae are characterized by five different craniofacial morphologies (Peterson et al. 2022). One such morphology is the blunt nose, which lacks facial protrusions. The tubesnout is aptly named for its distinct elongation of the frontal bones and the small pincer-like jaws that sit at the anterior-most tip of the rostrum. The Schnauzenorgan is a fleshy, highly mobile appendage that extends from the lower jaw (Fig. 2C). Some mormyrids have a tubesnout combined with a Schnauzenorgan (Fig. 2D and E), while other mormyrids have a chin swelling, a fleshy mass, that slightly protrudes from the lower jaw and superficially resembles a truncated Schnauzenorgan (Fig. 2A and B). Previously, these characters have been used for taxonomic assignments and generic descriptions, but in light of new molecular phylogenetic studies, the diagnostic utility of these morphological characters has become increasingly muddled. Peterson et al. (2022) showed that the genus *Marcusenius*, defined by a chin-swelling, is non-monophyletic; additionally, the genus *Gnathonemus* characterized by the Schnauzenorgan is non-monophyletic. Therefore, it was hypothesized that the diverse array of rostral appendages (Figs. 2 and 3) has evolved independently within multiple mormyrid lineages and may enhance electrosensory abilities and prey capture (Lavoué et al. 2012; Peterson et al. 2022).

Peterson et al. (2022) inferred a robust phylogeny for mormyrids and used ancestral state reconstruction to understand the pattern and timing of evolution of each craniofacial morphology. That study showed that three of the five different morphologies evolved multiple times, providing evidence for their adaptive value. The tubesnout has evolved up to four times in the genera *Mormyrus* and *Mormyrops* (Fig. 3, yellow) and is hypothesized to have evolved secondarily for active electrolocation and for facilitating the exploitation of novel trophic niches. The Schnauzenorgan and tubesnout are hypothesized to serve a similar function, that of probing the substrate in search of prey. However, this hypothesis is called into question as both a tubesnout and a Schnauzenorgan have evolved in *Gnathonemus echidnorhynchus* (Fig. 3, TS + SO1) and within *Campylomormyrus* (Fig. 3, TS + SO2) up to three times. The function of the chin swelling is unknown, but based on its external appearance, it could represent a precursor to a Schnauzenorgan or a tubesnout with a Schnauzenorgan. The chin swelling has evolved six times, two of which were in the species *Marcusenius livingstonii* (Fig. 3, CS1) as well as the true *Marcusenius* clade (Fig. 3, CS2). Based on Peterson et al. (2022) the blunt nose is the ancestral state of Mormyridae. Lastly, the Schnauzenorgan evolved once in the true *Gnathonemus* clade (*G. petersii* and *G. longibarbsis;*Fig. 2C; Peterson et al. 2022).

Despite having arisen only once, the Schnauzenorgan has been of great interest to anatomists given its adaptive function in enhancing the electrosensory ability of mormyrid fishes (Castelló et al. 2000; Bacelo et al. 2008; Caputi et al. 2011; Amey-Özel et al. 2015; Amey-özel 2018). Previous studies have demonstrated that the Schnauzenorgan functions as a probe that is densely covered in electroreceptors (Amey-Özel et al. 2015; Amey-özel 2018). The dense covering of electroreceptors has led to the Schnauzenorgan being classified as an area with high electrosensory acuity termed an electrosensory fovea (Bacelo et al. 2008; Amey-Özel et al. 2015; Amey-özel 2018; Caputi et al. 2011; Caputi and Budelli, 2006; Castelló et al. 2000). The probing movements are achieved by a modified posterior intermandibular muscle and two muscles of unknown origin that insert into a supportive rod made of mucochondroid tissue (Mikuriya 1972; Benjamin 1988; Amey-özel 2018). Previous investigation of mormyrid rostral appendages has added a great wealth of insight into how these fishes feed and navigate their environments with the aid of electroreception, yet these studies have focused on the anatomy of a single rostral appendage (Mikuriya 1972; Amey-özel 2018) or focused on the macroscopic convergence of rostral appendages. Importantly, few studies have considered how these structures might differ in anatomy, microstructure, and function given that they arose independently across mormyrids.

This study analyzes histological data in order to understand the microscopic features for three rostral appendages found within Mormyridae (chin-swelling: *M. ntemensis* CS1 and True *Marcusenius* clade CS2; Schnauzenorgan: *G. petersii* SO1; Tubesnout with a Schnauzenorgan: *G. echidnorhynchus* TS + SO1, and *Campylomormyrus* TS + SO2). We hypothesize that macroscopically convergent rostral appendages are also histologically convergent. To address this, we compare the histological features of the three different appendages. Lastly, we propose the function of the different rostral appendages based on muscular and connective tissue anatomy and discuss whether the chin swelling may represent an incipient Schnauzenorgan.

## Material and methods

### Sample collection

We obtained ethanol-preserved, formalin-fixed specimens from the Cornell Museum of Vertebrates and the American Museum of Natural History. Due to the rarity of mormyrid specimens in both museum collections and the aquarium trade, we were only able to obtain a total of 11 specimens representing six mormyrid species (Table 1). The taxonomic sampling included two independent gains of the chin swelling, two independent gains of the tubesnout with a Schnauzenorgan, and the single gain of a Schnauzenorgan (See Figs. 2 and 3).

**Table 1:** List of taxa examined in Chapter 4 including the catalog number and number of specimens sectioned.

Taxa	Catalog number	Number of specimen
*Marcusenius ntemensis*	CUMV 81,633	2
*Marcusenius livingstonii*	CUMV 93,900	2
*Gnathonemus petersii*	TBD UMMZ	2
*Gnathoemus echidnorhynchus*	CUMV 96,736	2
*Campylomormyrus phantasticus*	AMNH 249,843	2
*Campylomormyrus rhynchophorus*	AMNH 236,884	1

### Histology

Specimens were prepared for embedding by removing the appendage of interest. For individuals possessing both a tubesnout and a Schnauzenorgan, only the Schnauzenorgan was removed. If the Schnauzenorgan was longer than the Paraplast-block mold, the appendage was cut in half or thirds. Tissues underwent an ethanol dehydration series from 70% EtOH to 100% EtOH solutions. Following dehydration, tissues were immersed in xylene, a clearing agent, until translucent. The tissues were then placed in a solution containing a xylene and Paraplast mixture until they were completely infiltrated with Paraplast. The tissues were kept in a 100% Paraplast solution overnight in a vacuum oven at 56–59**°**C to ensure full penetration of Paraplast into the tissue. Finally, the tissues were oriented for cross-section in block molds and covered with fresh Paraplast. The resulting blocks were sectioned on a Microm microtome (HM 315) at a thickness of 7 µm, and slides were stained with hematoxylin and eosin.

## Results

### Histology of the chin swelling: *M. ntemensis* (CS1)

**Fig. 4:**
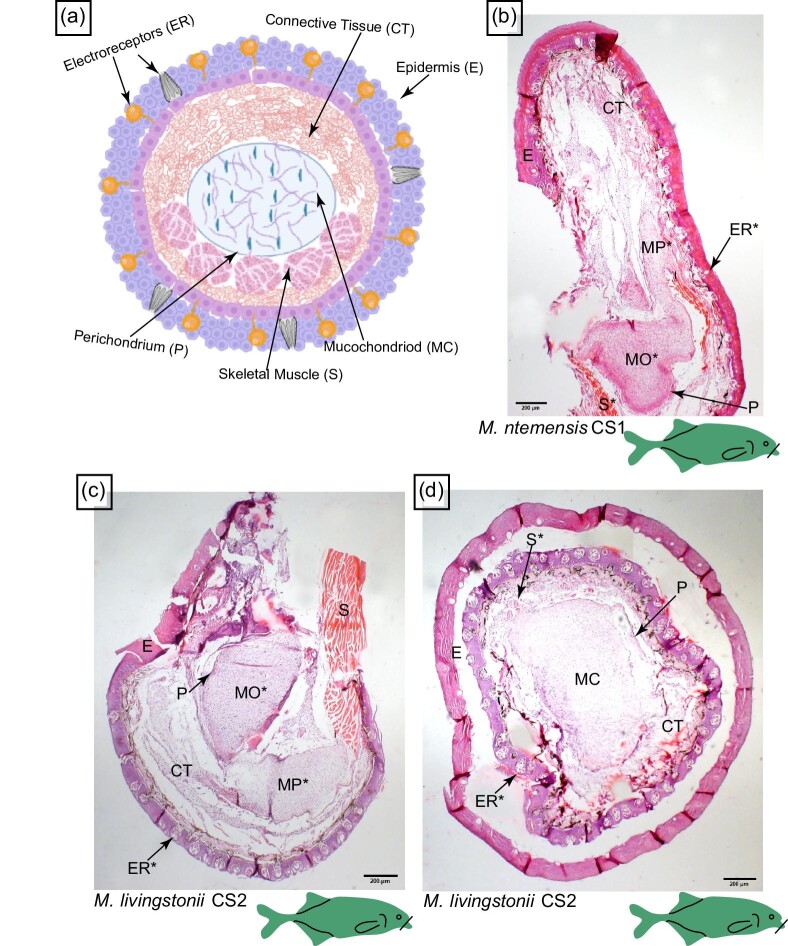
Histology of the chin-swelling in *M. ntemenis* and *M. livingstonii*. The line on the cartoon fishes indicates the location of the histological section shown and the * indicates anatomy that will be highlighted in Figure 5 and Figure 10. (A) Schematic representing the histology of the chin-swelling. (B) Cross-section of the chin-swelling of *M. ntemensis* (CS1) stained with H&E. (C and D) Cross-section of the chin-swelling of *M. livingstonii* (CS2) stained with H&E. E, epidermis; CT, connective tissue; MC, mucochondroid core; P, perichondrium; S, skeletal muscle; and MP, mucochondroid point.

**Fig. 5:**
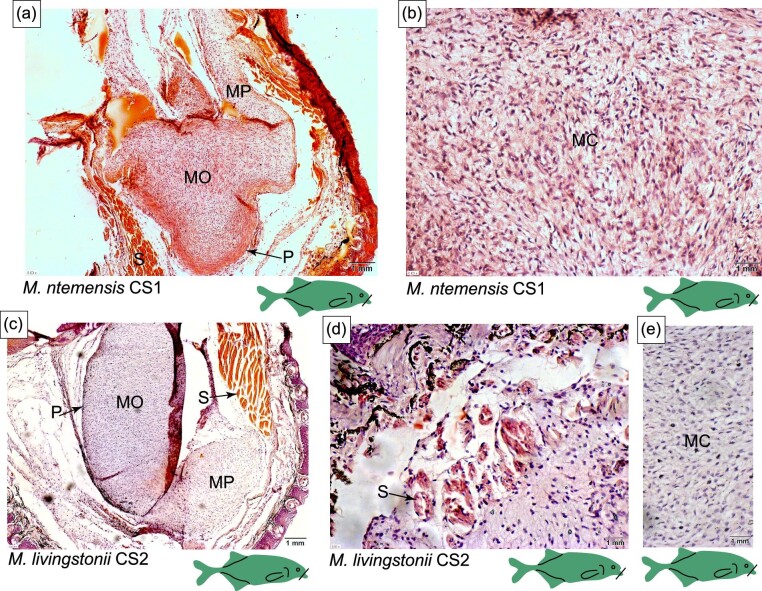
Histology of the chin-swelling in *M. ntemenis* and *M. livingstonii*. The line on the cartoon fishes indicates the location of the section shown. (A) Cross-section of the chin-swelling of *M. ntemensis* (CS1) stained with H&E, showing the two discrete oval-shaped (MO) and point-shaped (MP) components of the mucochondroid core. The perichondrium is indicated by the P. (B) Cross-section of the chin-swelling of *M. ntemensis* (CS1) highlighting the composition of the mucochondroid (MC). (C) Cross-section of the chin-swelling of *M. livingstonii* (CS2) stained with H&E. The perichondrium is designated by the P, and the two oval-shaped and point-shaped components of the mucochondroid are indicated by MO and MP respectively. (D) Cross-section of the chin-swelling of *M. livingstonii* (CS2) stained with H&E and skeletal muscle is shown by the letter S. (E) Cross-section of the chin-swelling of *M. livingstonii* (CS2) stained with H&E showing the composition of the mucochondroid (MC).

The chin swelling in CS1 *M. ntemensis* (Fig. 4B) is supported by a mucochondroid core. Adjacent to the mouth, the mucochondroid core has a complex architecture with two discrete parts that are oval- and point-shaped (see MO and MP in Figs. 4B and 5A). Within the mucochondroid core are densely packed fibroblast cells and collagen fibers with a thick perichondrium surrounding the core (see P in Figs. 4B and 5A). Surrounding and inserting into the mucochondroid core is darkly stained, longitudinal skeletal muscle (See S in Fig. 5A). Based on location and previous research, this muscle is likely the posterior intermandibularis (Mikuriya 1972). Embedded in the epidermis are electroreceptors (Figs. 4B and 10A), the most abundant type of which are mormyromasts (Fig. 10A). Based on previous research, mormyromasts are specialized for active electrolocation and easily identified by two sensory A cells that surround an empty outer chamber, connecting to the inner chamber which houses the sensory B cells (Bell et al. 1989). In addition to mormyromasts, we observed other electroreceptors but cannot definitively identify them due to the lack of diagnostic characters in the sections.

### Histology of the chin swelling: *M. livingstonii* (CS2)

Distal to the mouth, the mucochondroid core of the chin swelling in *M. livingstonii* (CS2) is oval shaped (see MC in Fig. 4D). More proximal to the mouth, the mucochondroid core has two discrete components that are oval and point shaped (see MO and MP in Figs. 4C and 5C). Compared to *M. ntemensis* CS1, the mucochondroid in *M. livingstonii* (CS2) has fewer fibroblasts and collagen fibers (Fig. 5B versus 5E). The composition of the perichondrium is consistent with that of *M. ntemensis* CS1 (P in Fig. 4B–D). Closer to the mouth, the chin swelling in *M. livingstonii* (CS2) has a band of skeletal muscles that inserts into mucochondroid. Distal to the mouth, the skeletal muscle is arranged in bundles dispersed within the connective tissue (see S in Figs. 4C, D and 5C, D). The electroreceptors present in the chin swelling of *M. livingstonii* (CS2) include mormyromasts, which are the most abundant, and other electroreceptors that cannot be characterized definitively (Fig. 10B). The epidermal layers separated during the embedding and sectioning process, leading to the outer chamber of the mormyromast splitting from the A and B cells (Fig. 10B).

### Histology of the Schnauzenorgan

**Fig. 6:**
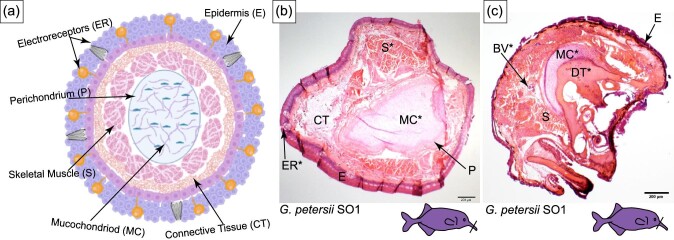
Histology of the Schnauzenorgan in *G. petersii*. The line on the cartoon fishes indicates the location of the section shown and the * indicates anatomy that will be highlighted in Figure 7 and Figure 10. (A) Schematic of the histology of the Schnauzenorgan in *Gnathonemus petersii*. (B and C) Cross-section of the Schnauzenorgan of *Gnathonemus petersii* (SO) stained with H&E. E, epidermis; CT, connective tissue; BV, blood vessel; MC, mucochondroid core; P, perichondrium; S, skeletal muscle; and DT, dentary.

**Fig. 7:**
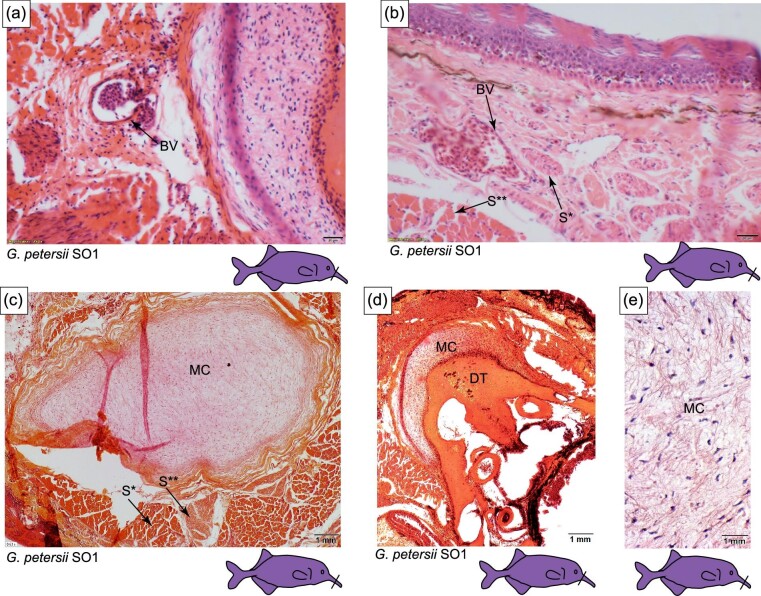
Histology of the Schnauzenorgan in *G. petersii*. The line on the cartoon fishes indicates the location of the section shown. (A) Cross-section of the Schnauzenorgan of *Gnathonemus petersii* (SO) stained with H&E highlighting the blood vessel (BV). (B) Cross-section of the Schnauzenorgan of *Gnathonemus petersii* (SO) stained with H&E highlighting BV and indicating the wider diamater skeletal muscle (S^**^) and the smaller diameter skeletal muscle (S*). (C) Cross-section of the Schnauzenorgan of *Gnathonemus petersii* (SO) stained with H&E showing the wider diameter skeletal muscle (S^**^) and the smaller diameter skeletal muscle (S*). (D) Cross-section of the Schnauzenorgan of *Gnathonemus petersii* (SO) stained with H&E showing the mucochondroid core (MC) and the dentary (DT). (E) Cross-section of the Schnauzenorgan of *Gnathonemus petersii* (SO) stained with H&E showing the composition of the mucochondroid core.

The Schnauzenorgan of *G. petersii* SO1 (true *Gnathonemus* clade) has an oval-shaped mucochondroid core distal to the mouth (see MC in Fig. 6B), while proximal to the mouth the mucochondriod is cup shaped and adjacent to a cup-shaped bone (see MC and DT in Fig. 6C). Based on location, the cup-shaped bone is likely the dentary. As seen in the chin swelling, the perichondrium in the Schnauzenorgan surrounds the mucochondroid (P in Fig. 6B). Around the entire mucochondroid are darkly stained longitudinal skeletal muscles that are most likely the posterior intermandibularis (S in Figs. 6B, C and 7B, C). Dispersed within the connective tissue are large blood vessels and lightly stained longitudinal skeletal muscles (BV in Fig. 7A and B). The darker stained skeletal muscle fibers are more abundant and have a wider diameter than the lighter stained skeletal muscle fibers (S in Fig. 7B and C). In the epidermis, mormyromasts are present along with other electroreceptors that cannot be characterized (Fig. 10C).

### Histology of the Schnauzenorgan arising from the tubesnout: *G. echidnorhynchus* (TS + SO1)

**Fig. 8:**
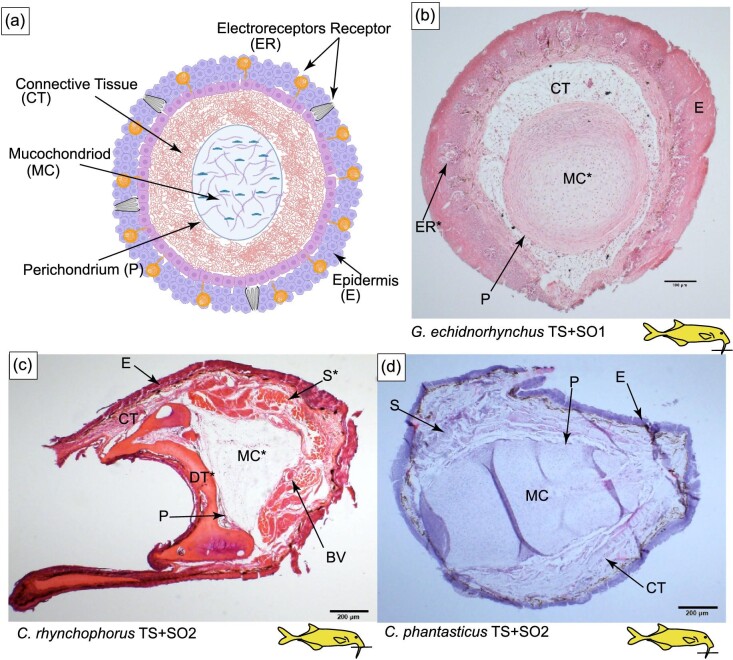
Histology of the tubesnout with a Schnauzenorgan in *G. echindnorhynchus, C. rhynchophorus*, and *C. phantasticus*. The line on the cartoon fishes indicates the location of the section shown and the * indicates anatomy that will be highlighted in Figure 9 and Figure 10. (A) Schematic of the histology of the tubesnout with a Schnauzenorgan. (B) Cross-section of the tubesnout with a Schnauzenorgan of *Gnathonemus echidnorhynchus* (TS+SO1) stained with H&E. (C) Cross-section of the tubesnout with a Schnauzenorgan of *Campylomormyrus rhynchophorus* (TS+SO2) stained with H&E. (D) Cross-section of the tubesnout with a Schnauzenorgan of *Campylomormyrus phantasticus* (TS+SO2) stained with H&E. E, epidermis; CT, connective tissue; MC, mucochondroid core; P, perichondrium; S, skeletal muscle; DT, dentary; and BV, blood vessel.

**Fig. 9:**
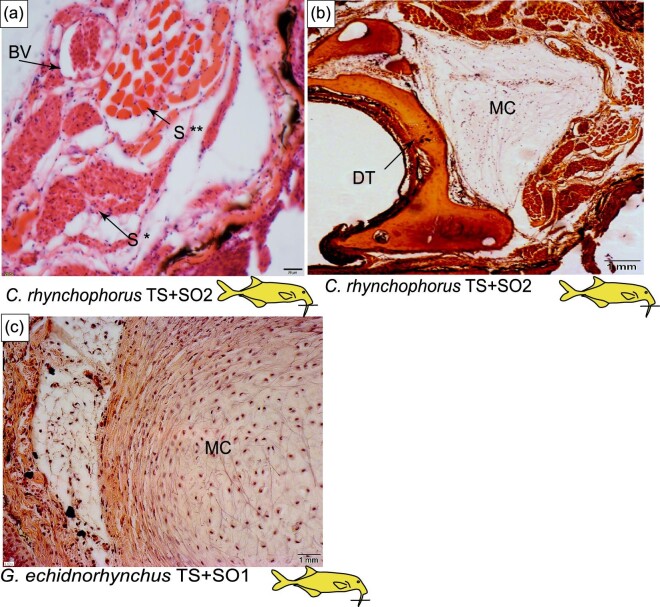
Histology of the tubesnout with a Schnauzenorgan in *C. rhynchophorus*, and *G. echindnorhynchus*. The line on the cartoon fishes indicates the location of the section shown. (A) Cross-section of tubesnout with a Schnauzenorgan of *Campylomormyrus* stained with H&E highlighting the blood vessel (BV) and the wide-diamteter skeletal muscle S^**^ and the small diameter skeletal muscle S*. (B) Cross-section of tubesnout with a Schnauzenorgan of *Campylomormyrus* stained with H&E indicating the dentary (DT) and the mucochondroid core (MC). (C) Cross-section of tubesnout with a Schnauzenorgan of *Campylomormyrus* stained with H&E.

**Fig. 10:**
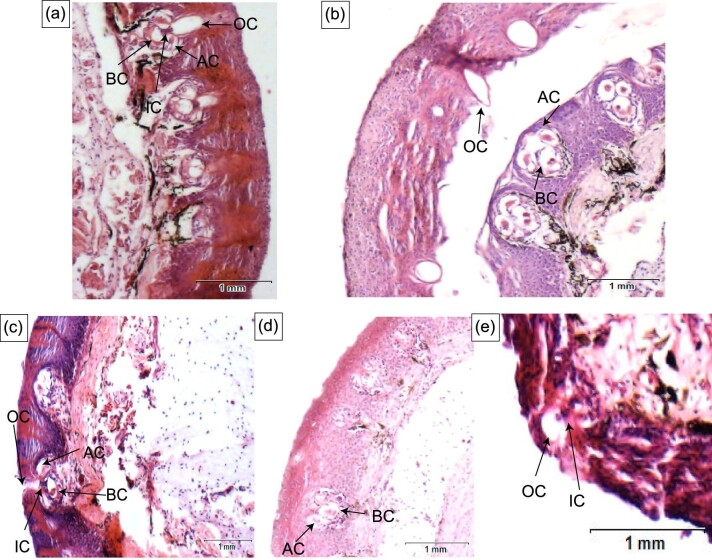
Histology of mormyromasts within Mormyridae: (A) Cross-section of the chin-swelling of *Marcusenius ntemensis* (CS1) highlighting the mormyromast. (B) Cross-section of the chin-swelling of *Marcusenius livingstonii* (CS2) highlighting the mormyromast. (C) Cross-section of the Schnauzenorgan of *Gnathonemus petersii* (SO) highlighting the mormyromast. (D) Cross-section of the tubesnout with a Schnauzenorgan of *Gnathonemus echidnorhynchus* (TS+SO1) highlighting the mormyromast. (E) Cross-section of the tubesnout with a Schnauzenorgan of *Campylomormyrus rhynchophorus* (TS+SO2) highlighting the mormyromast. OC, outer chamber; AC, A-cells; IC, inner chamber, BC, B-cells.

The tubesnout with a Schnauzenorgan as seen in *G. echidnorhynchus* (TS + SO1) has a circular mucochondroid core with densely packed fibroblast cells and collagen fibers (MC in Figs. 8B and 9C). Skeletal muscle was not observed: instead, loose connective tissue encircles the perichondrium (Figs. 8B and 9C). Embedded in the epidermis are mormyromasts and other electroreceptors (Fig. 10D).

### Histology of the Schnauzenorgan arising from the tubesnout: *Campylomormyrus* (TS + SO2)

In the tubesnout with a Schnauzenorgan of *Campylomormyrus* (TS + SO2), the mucochondroid core is oval shaped with evenly spaced fibroblast cells and a thick perichondrium (MC in Fig. 8D). Similar to the Schnauzenorgan in *G. petersii* (SO1), the rostral appendage of *Campylomormyrus* has two distinct components: a cup-shaped mucochondroid and a cup-shaped bone. These two distinct components are present adjacent to the mouth and may represent the point of attachment to the dentary (MC and DT in Figs. 8C and 9B). Surrounding three-fourths of the mucochondroid are lightly stained skeletal muscles with wide-diameter myofibrils interspersed with the less abundant darkly stained small-diameter myofibrils (S in Fig. 9A). Neighboring the skeletal muscles are loose connective tissue and large blood vessels. In the epidermis, we detected mormyromasts (Fig. 10E).

## Discussion

### Are independent gains of rostral appendages convergent?

Based on histological evidence, we show that independent origins of rostral appendages are convergent both macroscopically and microscopically. Both convergent incidences of the chin swelling (*M. ntemensis*, CS1, and *M. livinginstonii*, CS2) have similar anatomies that are composed of a central mucochondroid core that is surrounded by a perichondrium and skeletal muscle that surrounds half of the mucochondroid core. Surrounding the rest of the structure is connective tissue. Lastly, the epidermis of the chin swelling is embedded with densely packed electroreceptors (Fig. 10A and B). Similar to that of the chin swelling, the independent gains of the tubesnout with a Schnauzenorgan (*G. echidnorhynchus*, TS + SO1, and *Campylomormyrus*, TS + SO2) are convergent at a histological level. Both consist of a mucochondroid core covered by a perichondrium which is further surrounded by connective tissue. The epidermis is studded with electroreceptors (Fig. 10D and E). We did not, however, observe any skeletal muscle in the Schnauzenorgan of *G. echidnorhynchus* (TS + SO1) (Fig. 8B). In addition to convergent rostral appendages having similar microscopic anatomies, we show that *all* rostral appendages are composed of a mucochondroid core that is encircled by a thick perichondrium, connective tissue, and have an epidermis studded with electroreceptors. Despite these similarities, we find subtle anatomical differences, such as skeletal muscle distribution and the attachment shape of the appendage to the dentary. These anatomical differences likely contribute to the nuanced functional roles that these structures serve in locating prey within the substrate and enhancing electrosensory capabilities.

### Do all rostral appendages serve as a foveas?

A fovea is a specialized zone with (1) a higher density of receptor organs, (2) a central magnification (the area in the brain dedicated to processing a stimulus) of the brain region associated with the foveal region, (3) morphological specialization that enhances resolution, and (4) specific behaviors that are implemented to align the fovea with objects of interest (Bacelo et al. 2008). Based on brain mapping, electrosensory imaging, quantification of electroreceptor density, and behavioral studies, the Schnauzenorgan in *G. petersii* fits all the qualifications for an electrosensory fovea (Harder 1968; Quinet 1971; Von der Emde and Schwarz 2002; Bacelo et al. 2008). However, the electrosensory capacity of rostral appendages beyond that of *G. petersii* has been largely unexplored.

Two previous studies explored electroreceptor distribution across the body within mormyrids and reported a high density of mormyromasts and ampullary receptors within the nasal and chin regions (Quinet 1971; Hollmann et al. 2008). In congruence with previous studies, we show a high density of mormyromasts on the different rostral appendages (Fig. 10). Based on qualitative evaluation, compared to the Schnauzenorgan in *G. petersii*, the rostral appendages of *G. echindnorynhus* (TS + SO1), *M. livingstonii* (CS1), and *M. ntemensis* (CS2) all have a higher density of mormyromasts (Fig. 10). The high density of electroreceptors could be evidence that, like the Schnauzenorgan, the other rostral appendages also function as electrosensory foveas. We suggest that these rostral appendages are also foveas and show that they are morphologically specialized based on their histology in such a way that mormyrids are likely able to orient their appendages in the direction of objects of interest.

### Are rostral appendages muscular hydrostats?

Appendages that lack a skeletal system but are capable of highly controlled movements, elongation, shortening, bending, and torsion are called hydrostatic skeletons or muscular hydrostats (Chapman 1958; Kier and Smith 1985). A muscular hydrostat tends to be cylindrically shaped and characterized by three features: (1) muscles that are closely packed in a three-dimensional array (transverse, circular, or radial), (2) fibrous connective tissue, and (3) a liquid-filled structure that is a constant volume, such that a decrease in volume to one dimension of the muscular hydrostat results in an increase in another dimension (Clark and Cowey 1958; Kier 1992, 2016; Kier and Smith 1985).

We propose that the Schnauzenorgan in *G. petersii* (SO) and the tubesnout with a Schnauzenorgan in *Campylomormyrus* (TS + SO2) fit the diagnostic characteristics of a hydrostat. Both *Campylomormyrus* (TS + SO2) and G. petersii (SO) have densely packed longitudinal muscle fibers that encircle the mucochondroid and facilitate the movement of the appendage (Figs. 7C and 9A). Additionally, the presence of large blood vessels suggests they may play a role in keeping the appendage at a constant volume (Figs. 7A, B and 9A). While more testing is needed to confirm that the rostral appendages of *Campylomormyrus* (TS + SO2) and *G. petersii* (SO) are indeed muscular hydrostats, we hypothesize that they are highly mobile structures with the capacity to orient to objects of interest via elongation, shortening, bending, and torsion.

Due to a lack of blood vessels, we did not find histological evidence that the chin swellings could be muscular hydrostats. However, the histology does suggest that the chin swelling is capable of movement which may differ from that of other rostral appendages. We propose that the more skeletal muscle in a rostral appendage will result in a larger range of motion. Further, we hypothesize that the point-shaped mucochondroid likely functions similar to a hinge joint, capable of movement in one plane, while the cup-shaped dentary likely functions similar to a ball and socket joint capable of movement in all directions. Therefore, we propose that: (1) the Schnauzenorgan, with skeletal muscle completely surrounding the mucochondroid and a cup-shaped attachment point, is capable of movement in all directions; (2) the tubesnout with a Schnauzenorgan in *Campylomormyrus* (TS + SO1), with skeletal muscle surrounding three-fourth of the mucochondroid and a cup-shaped attachment point, is more limited in its range of motion while still capable of a hinging motion that flexes dorsally; (3) the chin swelling, with skeletal muscle surrounding half of the mucochondroid core and a point-shaped attachment point, has the most limited movement and is likely not capable of hinging motions; (4) the tubesnout with a Schnauzenorgan *G. echidnorhynchus* (TS + SO2; Fig. 8B), has no skeletal muscle which may indicate this appendage is fixed, but may also be an artifact of our limited sample size.

There are three types of skeletal muscle fibers: fast, intermediate, and slow twitch; differing compositions of these fibers produce movement in a range of speeds (Jayne and Lauder, 1994). A previous study used immunocytochemistry and enzyme histochemistry to identify muscle types as either fast, intermediate, or slow twitch, and showed that most fast-twitch fibers have a greater diameter than slow-twitch fibers (Hernandez et al. 2005). Within the rostral appendages of *G. petersii* and *Campylomormyrus*, there are two different types of skeletal muscle fibers present. Both wide-diameter and small-diameter skeletal muscles were discovered in the Schnauzenorgan of *G. petersii* (SO) and the tubesnout with a Schnauzenorgan of *Campylomormyrus* (TS + SO2), with the wide-diameter skeletal muscle present in greater abundance (Fig. 7B, C and Fig. 9A). Therefore, we hypothesize the Schnauzenorgan in *G. petersii* (SO) and the tubesnout with a Schnauzenorgan in *Campylomormyrus* (TS + SO2) are primarily composed of fast-twitch muscle fibers. Congruent with Amey-Ozel (2018), we propose that the fast-twitch muscle is responsible for total flexion while the less abundant slow-twitch muscle controls the constant probing motion. Fast and slow twitch muscles are thus likely present in the Schnauzenorgan (SO) of *G. petersii* (SO) and tubesnout with a Schnauzenorgan in *Campylomormyrus* (TS + SO2), but not in chin swellings. Given that the chin swelling experiences a much more limited range of motion, it is unsurprising that it also seems to experience a much smaller range of speeds at which it can move.

## Conclusions

Here, we present the first study to investigate the histological structure of independent gains of rostral electrosensory specializations within Mormyridae. At a histological level, we show that the convergent gains of rostral appendages share a similar tissue structure. Further, we find that all rostral appendages (i.e., chin-swellings and Schnauzenorgans) have similar histological architecture, while the differences in their anatomies likely relate to their range of mobility. Based on the different ranges of mobility, we propose these rostral appendages either function as a probe to manipulate the substrate, an electrosensory probe to enhance electrosensory abilities, or both. Using histological evidence alone, we demonstrate that the chin swelling and tubesnout with a Schnauzenorgan fit three of the four qualifications for an electrosensory fovea. Finally, the similar anatomy between the different appendages suggests that the chin swelling may be a precursor to Schnauzenorgan and a tubesnout with Schnauzenorgan.

## Data Availability

The data underlying this article will be shared on reasonable request to the corresponding author.
